# Creation of different bioluminescence resonance energy transfer based biosensors with high affinity to VEGF

**DOI:** 10.1371/journal.pone.0230344

**Published:** 2020-03-26

**Authors:** Constanze Stumpf, Tobias Wimmer, Birgit Lorenz, Knut Stieger

**Affiliations:** Department of Ophthalmology, Justus-Liebig-University Giessen, Giessen, Germany; Yale University School of Medicine, UNITED STATES

## Abstract

In age-related macular degeneration (AMD) or diabetic retinopathy (DR), hypoxia and inflammatory processes lead to an upregulation of the vascular endothelial growth factor (VEGF) expression and thereby to pathological neovascularisation with incorrectly formed vessels prone to damage, thus increasing the vascular permeability and the risk of bleeding and oedema in the retina. State of the art treatment is the repeated intraocular injection of anti-VEGF molecules. For developing improved individualized treatment approaches, a minimally invasive, repeatable method for in vivo quantification of VEGF in the eye is necessary. Therefore, we designed single molecule eBRET2 VEGF biosensors by directly fusing a Renilla luciferase mutant (Rluc8) N-terminal and a green fluorescent protein (GFP2) C-terminal to a VEGF binding domain. In total, 10 different VEGF biosensors (Re01- Re10) were generated based on either single domains or full length of VEGF receptor 1 or 2 extracellular regions as VEGF binding domains. Full length expression of the biosensors in HEK293-T cells was verified via Western Blot employing an anti-Rluc8-IgG. Expression of alternative splice variants was eliminated through the deletion of the donor splice site by introduction of a silent point mutation. In all ten biosensors the energy transfer from the Rluc8 to the GFP2 occurs and generates a measurable eBRET2 ratio. Four biosensors show a relevant change of the BRET ratio (ΔBR) after VEGF binding. Furthermore, each biosensor shows a unique detection range for VEGF quantification and especially Re06 and Re07 have a high sensitivity in the range of in vivo VEGF concentrations in the eye, previously measured by invasive methods. In conclusion, we generated several eBRET2 biosensors that are suitable for VEGF quantification in vitro and could identify two eBRET2 biosensors, which may be suitable for non-invasive in vivo VEGF quantification with an implantable device.

## Introduction

Besides uncorrected refractive errors and cataracts the predominant causes of visual impairment and blindness in the population over 50 years in high income countries are diseases like age-related macular degeneration (AMD), glaucoma and diabetic retinopathy (DR) [1]. During pathogenesis of all these diseases, changes in the vasculature of the retina play a crucial role, which is associated with an increased expression of the vascular endothelial growth factor (VEGF). Especially the homodimeric glycoprotein VEGF-A regulates vascular development and growth during vasculogenesis and angiogenesis and is important in AMD and DR pathogenesis. The VEGF family includes also VEGF-B, VEGF-C, VEGF-D, VEGF-E (found in viruses), VEGF-F (present in snake venom) and the placental growth factor (PLGF), which catalyses distinct functions dependent on the involved receptor [2].

Three VEGF receptors exist, which belong to the class of receptor tyrosine kinases (RTK). They have an extracellular ligand binding domain, that consists of seven immunoglobulin like domains and it is currently unclear, which of them are crucial for the VEGF binding. Some studies indicate that domain 2 and 3 of receptor 2 are important for the VEGF binding and that domain 3 is essential for the high affinity binding of VEGF-A [3–5]. Another group reported that the first three domains are necessary for the ligand binding, that the linker region between domain 2 and 3 of receptor 2 contains special residues crucial for the VEGF binding, and that the domains 4–7 reduce the overall binding affinity [6]. Furthermore, there are findings that a combination of the domains R1D2 and R2D3 lead to high affinity binding to VEGF with an optimized dissociation constant [7, 8]. In general ligand binding at the extracellular domain leads to the autophosphorylation of the receptor dimer at a tyrosine-kinase domain, thus activating interacting proteins and signalling cascades [9]. VEGF-A is able to bind VEGF receptor 1, VEGF receptor 2 (KDR) and neuropilin. The VEGFR 1 and 2 are both mainly located on the surfaces of endothelial cells and responsible for their proliferation, migration, and survival during vasculogenesis and angiogenesis. VEGFR 1 is also involved in monocyte migration, haematopoiesis, and proliferation in tumour cells [9–11].

Since increased VEGF-A levels are implicated in the pathogenesis, the current state of the art treatment for AMD and DR is the repeated intravitreal injection of anti-VEGF drugs like Bevacizumab (Avastin^®^), Ranibizumab (Lucentis^®^) and Aflibercept (Eylea^®^). These three different molecules are designed to capture VEGF via its receptor binding site, thus preventing its binding to the cellular VEGF receptors and the activation of subsequent signalling cascades. Several clinical trials showed significant improvement in visual acuity and general quality of life [12]. The drawback of this therapy is the half-life of the anti-VEGF drugs, resulting in the necessity of repeated injections. This represents not only a financial burden, it also increases the risk of side effects like endophthalmitis or retinal detachment and also causes more stress to the mostly older patients.

**Fig 1:**
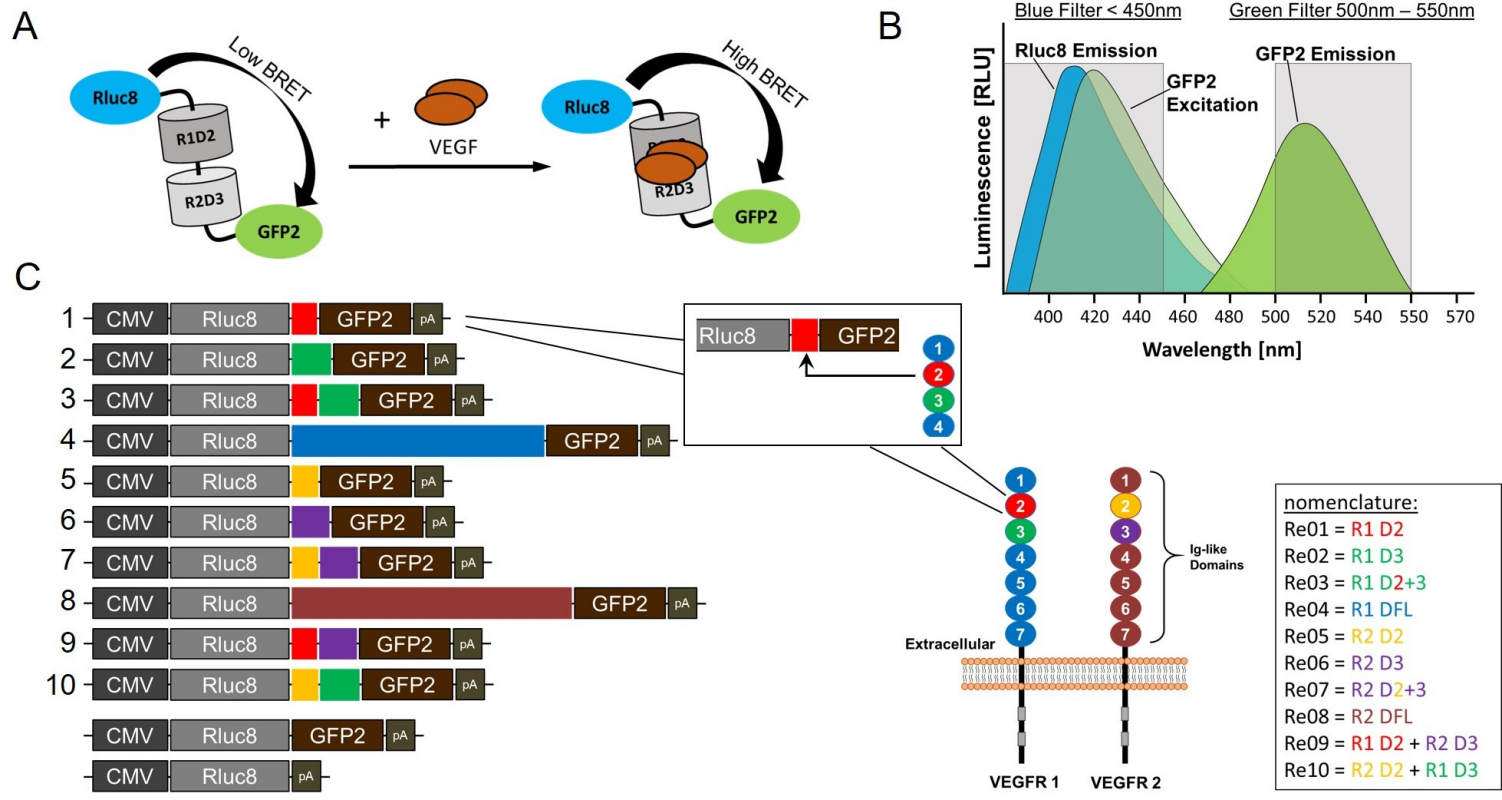
eBRET2 VEGF biosensors. (A) VEGF quantification method of the eBRET2 VEGF biosensor. A conformational change after VEGF binding leads to an increased energy transfer and thus to a higher BRET signal, in dependence of the VEGF concentration. (B) Overlapping Rluc8 emission wavelength and GFP2 excitation wavelength generates GFP2 emission at higher wavelength. (C) Biosensor expression constructs Re01–Re10. CMV = cytomegalovirus Promoter, Rluc8 = Renilla luciferase, R = VEGF receptor, D = IgG domain, GFP2 = green fluorescent protein, pA = polyadenylation signal. VEGF receptor 1 (R1) and 2 (R2) structure. The receptors contain a transmembrane-, an intracellular catalytic domain and 7 extracellular IgG like domains. IgG like domain 2 (D2) and 3 (D3) are responsible for the VEGF binding and were introduced between the Rluc8 and the GFP2 as VEGF binding domain.

Currently, there is no clinically approved and standardized method for measuring VEGF in the anterior chamber [13]. The evaluation of central retinal thickness (CRT) in spectral-domain optical coherence tomography (SD-OCT) scans and visual acuity data represent the current biomarker for treatment efficacy. Increase in CRT and a decline in visual acuity are indications for retreatment, but only occur with a delay of up to 4 weeks after the end of VEGF suppression [14]. In addition, the individual initial VEGF concentration and the individual VEGF suppression in each patient complicate the calculation of the best time point for the next anti-VEGF injection. Consequently, it would be desirable to detect the initial intraocular VEGF concentration with a non-invasive quantification method. The bioluminescence resonance energy transfer (BRET) is a modification of the fluorescence resonance energy transfer (FRET). The spectral separation of the fluorescent donor and acceptor limits the dynamic range of FRET based quantifications due to a low Stokes shift. Replacing the fluorescent donor of FRET systems with a bioluminescent donor (BRET) provides several advantages because photobleaching, autofluorescence and tissue attenuation due to fluorophore excitation are absent [15]. The spectral separation can be enhanced by replacing the donor fluorophore with a luminescent one (e.g. RLuc8) [16]. The increased separation of the donor and acceptor emission peaks (Rluc8 emits at ~ 400 nm and GFP2 emission peak lies at ~ 510 nm) of ~ 100 nm decreases the background signal which makes the eBRET2 variant one of the best BRET combinations [16–18]. The bioluminescent donor generates energy, which is transferred to a fluorescent acceptor molecule due to the spectral overlapping (resonance) of the donor emission wavelength and the acceptor excitation wavelength (Fig 1B). This energy transfer depends highly on the distance between the donor and the acceptor molecules and the parallelism of the dipole moments of the donor and the acceptor, so the 3-dimensional structure of the biosensor determines the quality of the energy transfer [19].

Quantifications with BRET can be performed in a one-molecule or a two-molecule approach. For setting up a two-molecule system, additional experiments like saturation assays to determine optimal donor/acceptor molecule ratios are needed, while one-molecule systems only require suitable controls (e.g.: correction factors [RLuc8] for BRET measurements and negative controls for quantification [19–21]. Interactions of the biosensor molecules with analytes, which are inducing are structural/conformational rearrangement can be used for quantification. The principle is a common tool for the observation of protein-protein interactions in cells and tissue [18, 22], for studies of receptor activation [23, 24], quantification of analytes [25, 26], drug residence time [27] and tumor imaging in vivo [28]. BRET systems can contain several different bioluminescent donors and different fluorophores as acceptor. We use the eBRET2 that combines the Rluc8 variant, a mutated Rluc optimized for enhanced stability and light emission, with the GFP2, and the luciferase substrate Coelenterazine 400a (CLZ400a). Usually the light output at the emission maxima of the luminescent donor and the fluorescent acceptor are measured with suitable transmission filters and the BRET ratio (Em_acceptor_/Em_donor_) is calculated and normalized with the correction factor (donor RLuc8) [26, 29].

Compared to FRET, contaminating light to excite the FRET donor fluorophore is absent in BRET, which enables the measurement of small eBRET2 ratio changes. Further, the ratiometric BRET measurement is decreasing the variability. Fluctuations in parameters like cell types, number of cells transfected/transduced, and assay volumes are reduced in this regard and membrane permeable, non-toxic luciferase substrates (CLZ400a) make BRET assays ideal for in-vivo and in-vitro assays [15, 16, 30, 31]. Alternative binding partners (e.g.: PEDF, VEGF-B, VEGF-C, VEGF-D, VEGF-E, PLGF, PDGF) [4, 6] of the inserted VEGF receptor domains have considered when using these biosensor construct in a relative complex environment like the eye. Therefore knowledge of the cytokine/protein profile in the eye is needed.

The BRET ratio itself will differ from cell line to cell line because the tertiary structure of the BRET biosensor will be different due to different posttranslational modifications. The fact that the energy transfer is highly dependent on the distance of the luciferase to the fluorophore and the parallelism of the dipols of the luciferase and the fluorophore. It has been shown that BRET systems are working in different cell types, ex vivo after purification in a microfluidic system and in vivo in several animal models [23,24,26,29].

The aim of this study was to generate an eBRET2 VEGF biosensor, which combines the best abilities in VEGF binding and simultaneous BRET performance. Therefore, we designed ten different eBRET2 VEGF biosensors, which contain only extracellular IgG like domain 2 of VEGF receptor 1 or 2, domain 3 of both receptors, domain 2 and 3 together of both receptors, the whole extracellular domain (full length) of both receptors and combinations of domains 2 and 3 of the receptors 1 and 2 similar to the aflibercept design (Fig 1C).

## Materials and methods

### Cell culture

HUVECs (human umbilical vein endothelial cells; ATCC:PCS-100-013, LGC Standards, Wesel) were cultivated in endothelial cell Medium M200 supplemented with Low Serum Growth Supplement (LSGS) Kit (both Thermo Fisher Scientific, Schwerte, Germany). HEK293T cells (ATCC® CRL-3216^™^) were maintained in DMEM (Dulbecco’s modified Eagle’s medium) supplemented with 1% penicillin/streptomycin, 4 mM L-glutamine and 10% fetal bovine serum (FBS) (all PAN Biotech, Aidenbach, Germany). Both celltypes were incubated at 37°C with 5% CO_2_ in a humidified incubator and sub cultured using Accutase.

### Extraction of the VEGF receptor domains

The amino acid sequence of the extracellular IgG Domains of VEGF receptor 1 and 2, described by [32], were taken to determine the correct position on the DNA level and Primer were designed for the domains 2, 3 and the full length extracellular domain without signal sequence (S1 Table). Seven million HUVEC cells were deployed to isolate total RNA with the RNeasy^®^ Mini Kit (Quiagen, Hilden, Germany). Then mRNA was transcribed into cDNA with the TaKaRa PrimeScript 1^st^ strand cDNA synthesis Kit (Takara Bio Inc., Kusatsu, Japan). The primers for receptor 2 were used in a PCR to amplify the domains 2, 3, 2 and 3 together and the full length from the cDNA. In a PCR with the receptor 1 primers the pcDNA3.1(+)-sFlt1 vector was used. This vector contains the soluble VEGF R1 isoform 1 that was generated via PCR from human corneal mRNA.

### Construction of the eBRET2-VEGF biosensors

The pcDNA3.1(+)-Rluc8-Ra02-GFP2 vector [19] was used as a Backbone. With the reverse primer in the Luciferase (5’→ 3’: CTGCTCGTTCTTACGCACGCG) and the forward primer in the GFP2 (5’→ 3’: AGCGGGGGCGAGGAGCTGTTC) the backbone was amplified via PCR without its VEGF binding domain (Ra02). The PCR amplified DNA of the receptor domains were introduced between Rluc8 and GFP2 in different combinations with a T4 DNA Ligase (New England Biolabs, Frankfurt a.M. Germany) to generate 10 new eBRET2 VEGF biosensors (Fig 1C). A pcDNA3.1(+)-Rluc8-GFP2 was used as a maximum control and a pcDNA3.1(+)-Rluc8 was used as the correction factor in the eBRET2 measurements. Both vectors were generated by the deletion of the VEGF binding domain and the GFP2 respectively via PCR.

### Transfection and cell lysis

HEK293-T cells were seeded in 6 well plates 24 h before transfection. Before transfection the culture media was replaced with fresh, supplemented DMEM (1.7 ml). Biosensor plasmids (6 μg each) and the controls, were added to 100 μl NaCl (150 mM) and mixed with 200 μl PEI Mix (0.1 g Polyethylenimine/L (MW: 25 kDa); 150 mM NaCl; Polyscience Inc., Warrington, USA). After 5 minutes incubation at room temperature the mixture was added to the cells. The Medium was changed again after 6 hours to avoid cell stress due to serum deprivation and polyethylenimine toxiciticy [33, 34]. The cells were incubated additional 24 h at 37°C with 5% CO2, then washed with phosphate buffered saline (PBS) and harvested with luciferase lysis buffer (Promega, Mannheim Germany) with a cell scraper. Lysis was maintained by two freeze/thaw cycles in liquid nitrogen, clarification of the lysate was carried out at 14000 g for 5 min. and 4°C. For further analysis the lysates were stored temporarily on ice.

### Fluorescence microscopy

24 h after transfection the activity of the biosensor component GFP2 was verified by fluorescence microscopy with the Keyence BZ-8000 (Keyence, Neu-Isenburg, Germany). Culture media was discarded and the cells were washed once with PBS to reduce the background signal of the Medium. Phase contrast images were recorded with an exposure time of 1/2 s and fluorescence images with < 1s exposure respectively.

### Luminescence measurements

The lysates of the biosensors and the controls were pipetted in triplicates into a 96 well plate (COSTAR Lumiplates Flat White; Corning, Berlin, Germany) with 20 μl per well. The Luciferase substrate Coelenterazine 400a (CLZ400a; NanoLight Inc., Pinetop, USA) with a concentration of 0.5 mg/ml in 100% ethanol was diluted 1:100 in eBRET2 assay buffer (PBS supplemented with 1 g/L D-glucosemonohydrate (Roth; Karlsruhe Germany), 0.1 g/L calciumchloridedihydrate (Merck; Darmstadt Germany) and 0.1 g/l magnesiumchloride-hexahydrate (Merck; Darmstadt Germany)) and were incubated 20 min. at room temperature in a lightproof tube. 100 μl of the substrate solution was added per well with the injector of the Tecan Infinite M1000Pro plate reader (Tecan; Groeding Austria) and readings were taken directly. The RLuc8 substrate (coelenterazine 400a) in BRET assay buffer was incubated for 20 min. at room temperature under light protection to avoid light emission caused by substrate oxidation. Nevertheless these emissions are usually very low, ranging from >0 to approx. 100 photons per second, making the substrate emission almost negligible. To determine the Luciferase activity luminescence measurements were taken with open filters and an integration time of 1 s.

### eBRET2 measurements

The lysates of the biosensors and the controls were also pipetted in triplicates into a 96 well plate with 20 μl per well. After substrate addition (see luminescence measurement) the single-molecule eBRET2 biosensor are generating two emission maxima at different wavelengths. For eBRET2 measurements the dual colour luminescence mode of the Tecan plate reader was used with two filter in the range of 370–450 nm (Blue filter) and 510–540 nm (Green filter). The measurements were carried out with an integration time of 1s.

### Calculation of eBRET2 Ratio

eBRET2 ratio was calculated after dual luminescence measurements via dividing the Green filter value by the Blue filter value (Eq 1) [19]. The mean eBRET2 ratio was computed using triplicate measurements with standard deviation. For normalization the eBRET2 ratio of the correction factor (cF) Rluc8, measured like the biosensor samples, was subtracted.

$$eBRET2\;Ratio=\frac{Biosensor\;(Green\;filter)}{Biosensor\;(Blue\;filter)}-\frac{cF\;(Green\;filter)}{cF\;(Blue\;filter)}$$ (1)

### eBRET2 ratio in dependence of the VEGF concentration

The biosensor concentrations were adjusted with luciferase lysis buffer to equal amounts on the basis of Rluc8 expression. 20 μl of adjusted biosensor lysates were incubated with 10 μl serially diluted recombinant human VEGF (Active Max^®^ VEGF-A165, Acro Biosystems, expressed in HEK293) at 4°C under gentle agitation over night. The samples were transferred onto a 96 well plate (Corning; Berlin, Germany) in triplicates and VEGF dependent eBRET2 ratios were observed via two filter luminescence measurement with the Tecan plate reader (Tecan; Groeding Austria) after injection of 100 μl substrate solution (CLZ400a 5 μg/ml) per well. Mean eBRET2 ratios were calculated and normalized with the corresponding biosensor (negative control) without VEGF (Delta eBRET2 ratio) (Eq 2) [30].

$$Delta\;eBRET2\;Ratio=\frac{Biosensor+VEGF\;(Green\;filter)}{Biosensor+VEGF\;(Blue\;filter)}-\frac{Biosensor-VEGF\;(Green\;filter)}{Biosensor-VEGF\;(Blue\;filter)}$$ (2)

### Western Blot analysis

Cell lysates with equal biosensor concentrations were used in SDS-Page to separate Proteins by size. Afterwards Proteins were transferred onto a nitrocellulose membrane (BioTrace^™^ NT, Pall Corporation; Port Washington, NY, USA) via semidry blotting system (Biometra; Göttingen, Germany) and the membranes were blocked for 1 hour in 3% milk/TBS. As first antibodies for the detection of the biosensors and the loading control GAPDH the polyclonal antibody rabbit anti-Rluc (Abcam; Cambridge, UK) and the monoclonal antibody rabbit anti-GAPDH respectively (Cell signaling; Danvers, MA, USA) were used. According to this the membranes were incubated with the second antibody goat anti-rabbit-IgG conjugated with horse radish peroxidase (HRP) (Sigma Aldrich; Taufkirchen, Germany). For HRP detection the GE Healthcare Amersham^™^ ECL^™^ Western Blotting-detection reagent and the chemiluminescent films GE Healthcare^™^ Amersham^™^ Hyperfilm ECL (GE Healthcare, Chicago, USA) were used.

### Test for splice sites

Several potential donor and acceptor splice sites within the expression cassettes of all biosensor variants were identified using the online tool Human Splicing Finder 3.0 [35]. To test for concrete splice sites HEK293-T cells were transfected with biosensor plasmids and harvested after 24 h. Using the RNeasy^®^ Mini Kit (Quiagen; Hilden, Germany) RNA was isolated and afterwards transcribed into cDNA (TaKaRa PrimeScript 1st strand cDNA synthesis Kit, Takara Bio Inc., Kusatsu, Japan). Biosensor expression cassettes in Plasmids and cDNA were amplified via PCR with a forward primer in the Rluc8 and a reverse Primer in the GFP2 and analysed using Agarose-Gel electrophoresis. The cDNA Bands were cut out and DNA was isolated with NucleoSpin® Gel Clean-up Kit (Marcherey- Nagel, Düren, Germany) and sent to Microsynth Seqlab (Göttingen, Germany) for Sanger sequencing.

### Deletion of the splice site

One detected strong donor splice site in the Luciferase was deleted via mutagenesis PCR which results in a nucleotide exchange within these splice site. Only one nucleotide was changed (T→ A) at the end of a triplet which didn’t shift the open reading frame or altered the amino acid sequence.

### Statistical testing

All data are presented as mean ± standard deviation (SD). For analysis of statistical relevant differences, data were compared with the students t-test using SigmaPlot (Systat Software; Erkrath, Germany) and the p < 0.05 was set as statistically significant. The p < 0.001 was set as statistically highly significant.

## Results

**Fig 2:**
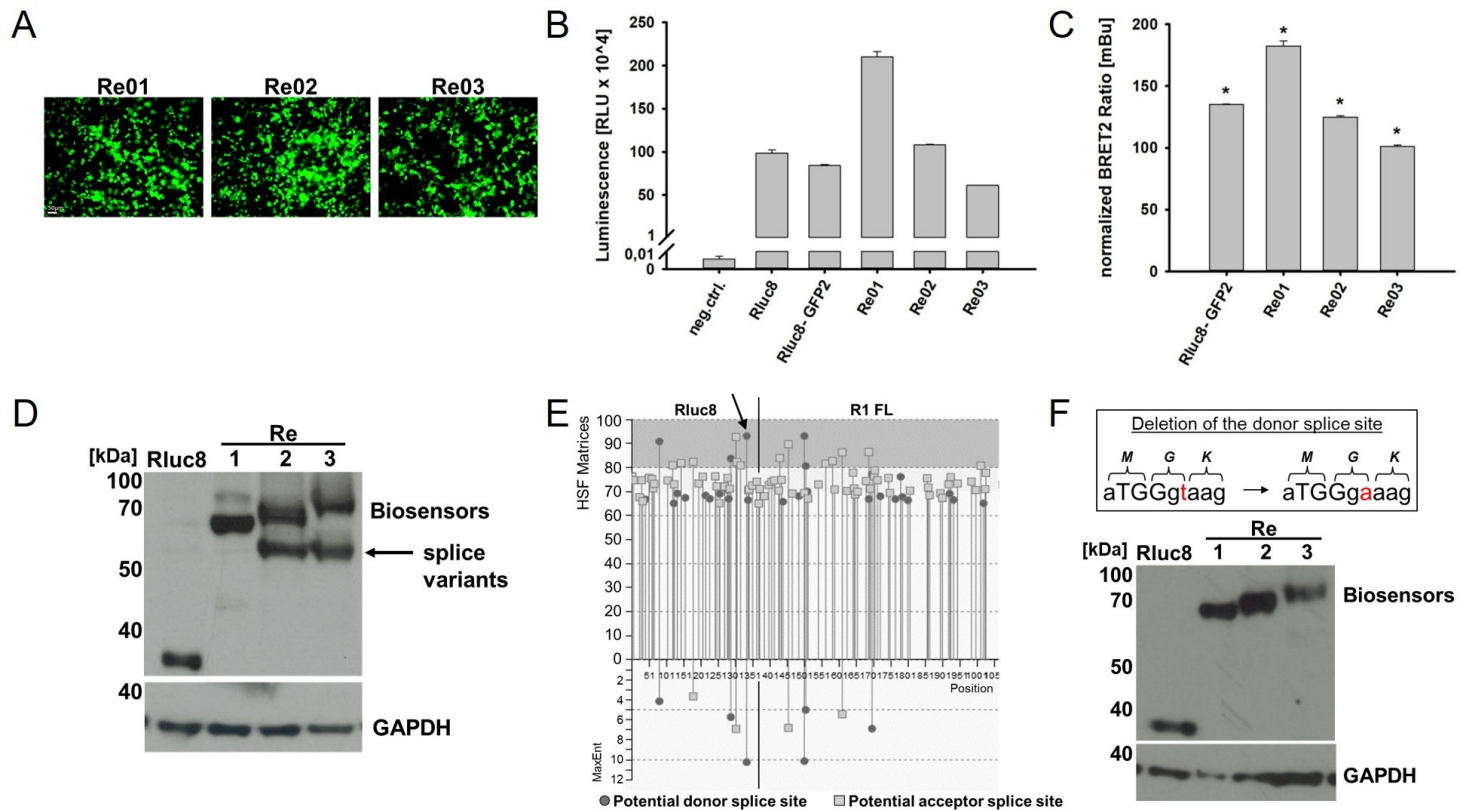
Characterization of eBRET2 biosensor expression. (A) Fluorescence microscopy of GFP2 expression after biosensor plasmid transfection in HEK293 T cells. (B) Luciferase activity via Luminescence measurement. Rluc8 expression correlates with Luciferase activity. (C) eBRET2 ratios of the biosensor variants normalized with the correction factor (Rluc8 29.8 ± 0.3 mBU). Significance defined as p < 0.001. (D) Western Blot shows full length expression of the biosensors and correction factor (Rluc8 alone). 1. Antibody: anti Rluc8 (1:1000) on the top and anti GAPDH (1:1000) on the bottom. 2. Antibody: anti Rabbit HRP (1:20000). Arrow indicates additional bands that show extra splice variants. (E) Splice prediction with Human Splicing Finder 3.0 software. Circles represent donor sites, squares represent acceptor sites, and strongest candidates are located in the grey area. Arrow marked the strong donor splice site in the Rluc8. (F) Western Blot after deletion of the splice site shows full length expression of the biosensors and correction factor (Rluc8 alone). No splice variants could be detected. The box shows the base sequence of the splice site with one letter code for the corresponding amino acids above. In red marked the change of the thymine base into an adenine base to delete the splice site. The amino acid sequence did not change.

Ten new eBRET2 VEGF biosensor variants were generated by introducing different VEGF binding domains based on the extracellular domains of the VEGF receptors 1 and 2, called Re01–Re10 (Fig 1C). The Rluc8-Ra02-GFP2 plasmid [19] served as backbone and the VEGF binding domain were replaced by the extracellular IgG like domains 2, 3, 2–3 and 1–7 (full length) from the VEGF receptors 1 and 2, respectively. Furthermore, combinations between the receptor domains 2 and 3 of both receptors were designed according to the anti-VEGF molecule Aflibercept [7] and vice versa. The Rluc8-GFP2 plasmid, in which the VEGF binding domain was deleted, served as maximum control. For normalization of eBRET2 measurements, an Rluc8 plasmid that only provides a luciferase signal, was used. HEK293-T cells were transfected with the biosensor plasmids and cultivated 24 h to allow the expression of the biosensors for the analysis of activity of the single components of the eBRET2 biosensors. Fluorescence microscopy showed for all ten biosensors the expression of an active GFP2 (Fig 2A). Subsequently, transfected HEK293-T cells were lysed and luminescence measurements with open filters were performed with the clarified lysate after the addition of the luciferase substrate Coelenterazine 400a. The negative control showed a light output of 57.67 ± 14.57 RLU whereas the correction factors provided outputs of 98.700 x 10^4^ ± 3.640 x 10^4^ RLU (Rluc8) and 84.026 x 10^4^ ± 1.482 x 10^4^ RLU (Rluc8-GFP2). Light outputs of the biosensors were 210.263 x 10^4^ ± 6.059 x 10^4^ RLU for Re01, 108.447 x 10^4^ ± 0.488 x 10^4^ RLU for Re02 and 62.288 x 10^4^ ± 0.326 x 10^4^ RLU for Re03 (Fig 2B).

In order to determine, whether the energy transfer from the Rluc8 to the GFP2 occurs, a luminescence measurement with two filters in the range of 370–450 nm (Blue filter) and 510–540 nm (Green filter) was performed with the clarified lysates of the transfected HEK293-T cells after Coelenterazine 400a addition. The mean eBRET2 ratio for every biosensor was calculated (Green filter/Blue filter) and normalized with the correction factor Rluc8 (29.8 ± 0.3 mBU). The calculated eBRET2 ratios were 182.4 ± 4.3 mBU, 124.8 ± 1.2 mBU, and 101.1 ± 1.3 mBU for biosensors Re01 to Re03, respectively (Fig 2C). All biosensors showed an eBRET2 ratio that was significantly different compared to the correction factor Rluc8 (p < 0.001).

To verify the full length biosensor expression, a Western Blot was performed with an anti-Rluc8 antibody and GAPDH used as loading control. All biosensors showed bands at their estimated size (e.g. Rluc8 at ~ 35 kDa, Re01 at ~ 68 kDa, Re02 at ~ 70 kDa, and Re03 at ~ 80 kDa). However, in addition to the expected bands, all biosensor samples showed an additional band at ~ 60 kDa (Fig 2D). These extra bands were similar in size as the Rluc8-GFP2 construct, providing some evidence that alternative splicing was taking place leading to the loss of the VEGF binding domain in some of the biosensor proteins. To proof this theory, the DNA sequences were analysed with the online tool Human Splicing Finder 3.0 [35] to identify potential donor and acceptor splice sites. The output of this tool revealed the presence of several potential splice sites within the open reading frame of all biosensors (Fig 2E). To identify major splice sites responsible for generating the additional protein in the Western Blot, the RNA of transfected HEK293-T cells was isolated, transcribed into cDNA and RT-PCR was performed. For all ten biosensors, alternative splice variants could be identified, which lost their VEGF binding domain because of a strong donor splice site at the end of the luciferase cDNA and different acceptor splice sites within the GFP cDNA or the VEGF binding domain cDNA. Consequently, these splice variants still contain an active luciferase and in most cases an active GFP2 but are not able to bind VEGF, thus disturbing the eBRET2 signal. In order to remove the donor splice site, one Thymine base in the consensus sequence was replaced by an Adenine base via PCR (Fig 2F). This silent mutation did not change the amino acid sequence. Again a Western Blot was performed and the splice variants were not detected anymore (Fig 2F).

**Fig 3:**
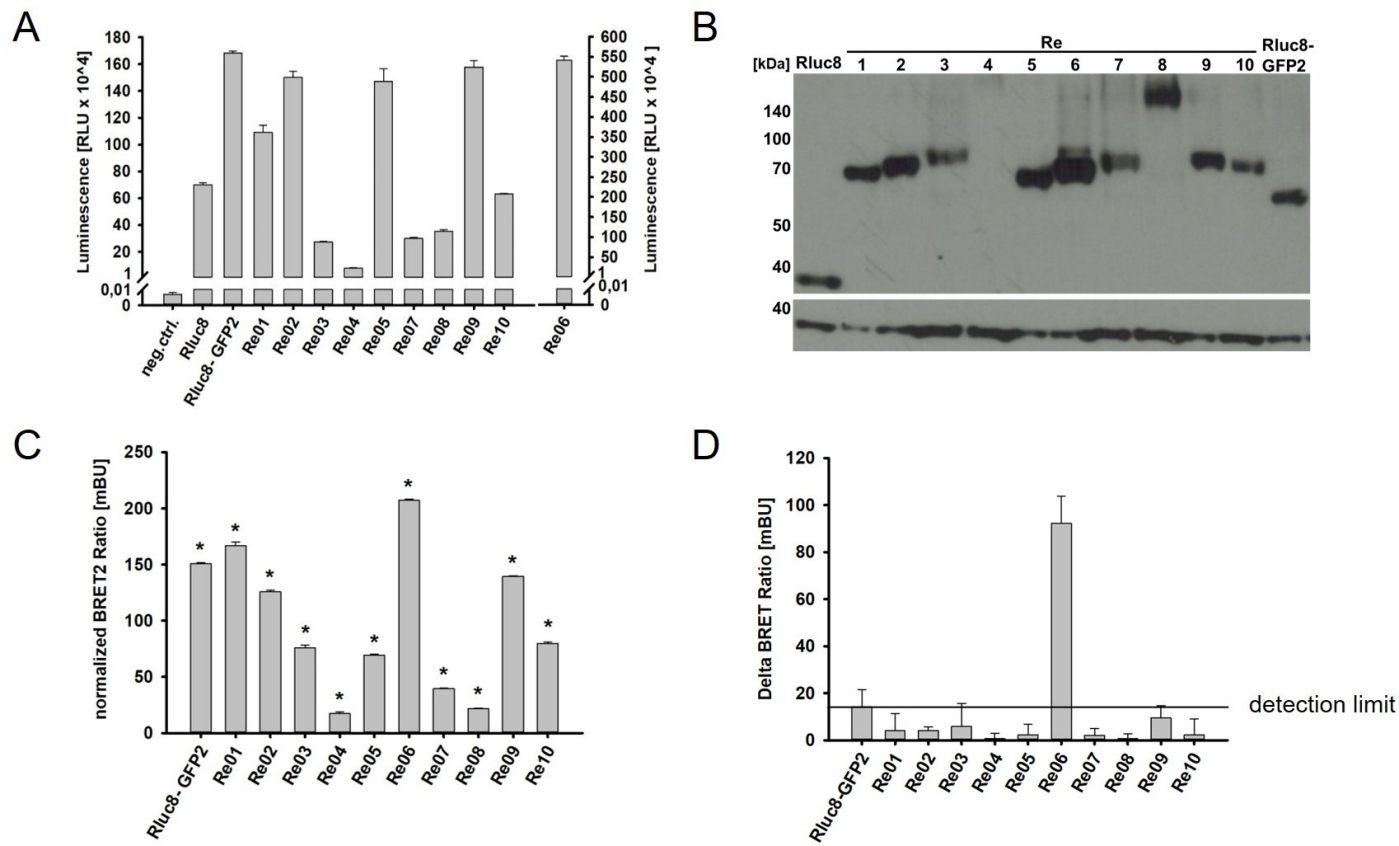
Characterization of eBRET2 biosensor expression after the deletion of the splice site. (A) Luciferase activity via Luminescence measurement. Rluc8 expression correlates with Luciferase activity. (B) Western Blot shows full length expression of the biosensors, correction factor (Rluc8 alone) and maximum control Rluc8 GFP2. 1. Antibody: anti Rluc8 (1:1000) on the top and anti GAPDH (1:1000) on the bottom. 2. Antibody: anti Rabbit HRP (1:12000). (C) eBRET2 ratios of the biosensor variants normalized with the correction factor (Rluc8 30.1 ± 0.1 mBU). Significance defined as p < 0.001. (D) Delta eBRET2 ratios after incubation with 10 ng VEGF. Change in ΔBR of the maximum control (Rluc8 GFP2) was set as detection limit, marked with the line.

After this codon optimization, the functionality tests for the single biosensor components were performed again. Transfected HEK293-T cells showed GFP2 fluorescence for all ten biosensors (S1 Fig) and luminescence measurements with the lysates showed a luciferase activity for all biosensors as well (Fig 3A).

Western Blot performed with lysates of transfected HEK293-T cells with an anti-Rluc8 antibody and GAPDH as loading control showed correct full length expression of the biosensors at the estimated sizes. This gives an overview of the differences in the dimensions of the biosensor proteins. Those with corresponding VEGF domains of the two receptors show similar sizes. Re01 and Re05 are the smallest with ~ 68 kDa, followed by Re02 and Re06 with ~ 71 kDa. Re03, Re07, Re09 and Re10 each contain two VEGF binding domains what lead to a size of ~ 80 kDa. The largest proteins are Re04 and Re08 with ~ 133 kDa and ~ 141 kDa which contain the full length extracellular domains of the VEGF receptors (Fig 3B).

In addition to a luminescence measurement with open filter and a Western Blot, a luminescence measurement with two filters (Blue and Green) was performed to calculate the eBRET2 ratio. Data were normalized with the correction factor Rluc8 (30.13 ± 0.10 mBU). Every biosensor showed a significantly different eBRET2 signal compared to the correction factor Rluc8 (p < 0.001) (Fig 3C).

To determine the changes of the eBRET2 ratios in the presence of VEGF, biosensor concentrations were set to equal amounts in the lysates by correlation with the luciferase activity, which is directly proportional to the biosensor concentration. The biosensors were incubated with VEGF at a concentration of 10 ng/ml for 24 h and two filter luminescence measurements were performed. Delta eBRET2 ratios (ΔBR) were calculated by normalization with the negative control (PBS). Statistical analysis showed that the two biosensors Re02 and Re06 generate a significant change in the eBRET2 ratio compared to the corresponding negative control (Re02: p < 0,003, Re06: p < 0,005). However, the control Rluc8-GFP2 shows a background ΔBR of 14.18 ± 7.37 mBU. Only Re06 passes the value, which indicates the technical deviation range, and thereby it is the only biosensor with a relevant change in ΔBR (Fig 3D).

Furthermore equal amounts of biosensors were incubated with different concentrations of VEGF in a range from 1 pg/ml to 10 ng/ml and ΔBR were calculated. The four biosensors Re02, Re06, Re07 and Re09 showed distinct changes in the ΔBR after VEGF binding. Interestingly, every biosensor showed the maximum change in ΔBR at another VEGF concentration (S2 Fig). Therefore, we further investigated the VEGF detection range of every biosensor and its dependence on the biosensor concentration. Three different biosensor concentrations (200,000 RLU, 400,000 RLU, and 800,000 RLU) were incubated with different VEGF concentrations and ΔBR was calculated.

**Fig 4:**
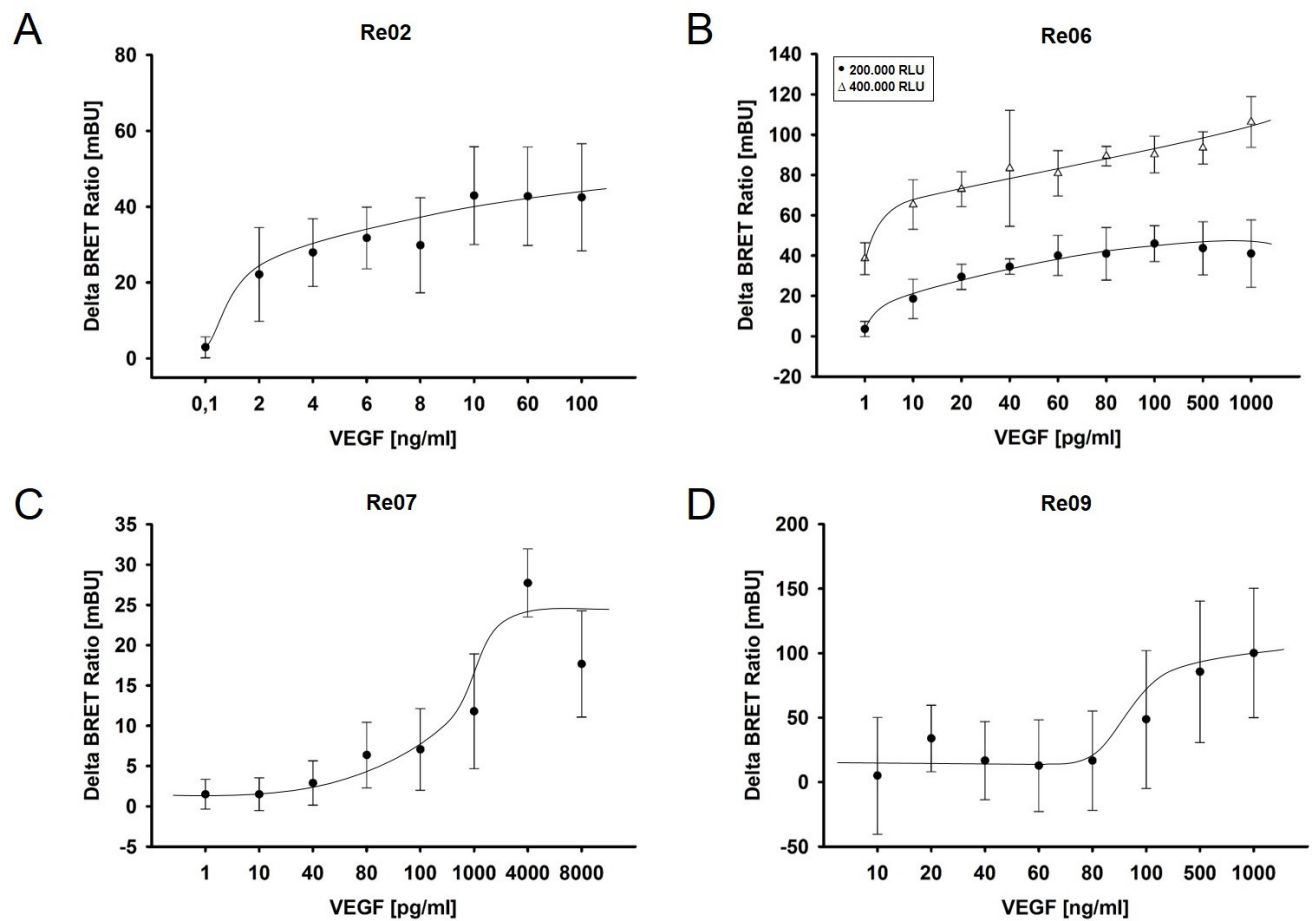
VEGF dependent Delta eBRET2 Ratios of the four biosensors Re02 (A), Re06 (B), Re07 (C) and Re09 (D). Biosensor concentrations in cell lysates were adjusted on the basis of RLuc8 expression to 200,000 RLU and 400,000 RLU. Lysates were incubated with individually adjusted VEGF concentrations and Delta eBRET2 Ratios were calculated. Significance defined as p < 0.05.

For Re02, a VEGF concentration range of 0.1–100 ng/ml was used. The highest change in ΔBR was observed at 200,000 RLU (Fig 4A). At 0.1 ng/ml VEGF the ΔBR was 2.95 ± 2.75 mBU whereas at 100 ng/ml the ΔBR was 42.48 ± 14.16 mBU. Starting from 4 pg/ml the changes in ΔBR were statistical significant compared to the control without VEGF (p < 0.05). Re06 was incubated with VEGF in a range of 1–1000 pg/ml and the highest ΔBR was observed at 400,000 RLU. Re06 had its maximum ΔBR 106.32 ± 12.6 mBU at a VEGF concentration of 1000 pg/ml whereas the ΔBR at this VEGF concentration with 200,000 RLU was at 40.97 ± 16.73 mBU. Compared to 400,000 RLU, which showed a significant change in ΔBR at 1 pg/ml, the 200,000 RLU showed a significant change in ΔBR at 20 pg/ml compared to the control without VEGF (p < 0.05). Re07 was incubated at 400,000 RLU with VEGF in a range of 1 pg/ml to 10 ng/ml and had is maximum ΔBR with 27.73 ± 4.23 mBU at a concentration of 4 ng/ml VEGF (Fig 4C). Re09 also showed an impact of biosensor concentration on ΔBR. The highest biosensor concentration (800,000 RLU) had a maximum ΔBR with 100.12 ± 50.15 mBU at a VEGF concentration of 1 μg/ml (Fig 4D). The biosensor concentration of 200,000 RLU also showed the maximum ΔBR at this VEGF concentration, albeit with a maximum change of 62.59 ± 15.09 mBU. Re09 shows a statistical significant change in ΔBR at a VEGF concentration of 500 ng/ml compared to the control without VEGF (p < 0.05).

## Discussion

To optimize an individualized therapy for patients with retinal or choroidal vascular diseases who were treated with anti-VEGF medications, the availability to quantify VEGF before and after anti-VEGF treatment would be advantageous. Wimmer et al. introduced a eBRET2 based VEGF biosensor as a promising tool for in vitro and in vivo quantification of VEGF [19]. In the present study, we wanted to further optimize such eBRET2 based VEGF biosensors by replacing the Ranibizumab based VEGF binding domain with a VEGF receptor based strategy.

There are three major requirements that should be fulfilled to generate a functional and efficient eBRET2 based biosensor. First, the emission wavelength of the donor and the excitation wavelength of the acceptor have to overlap. Second, the distance between the donor and the acceptor molecule should be less than 10 nm, and third the relative orientation of the donor and the acceptor should allow a non-radiative dipole-dipole coupling for sufficient energy transfer [36]. To address the first requirement, the spectral overlap for the eBRET2 pair Rluc8 and GFP2 was shown previously [37]. The second and third requirement need to be analysed for every new biosensor variant separately, because the change of the VEGF binding domains may alter the sterical performance of the molecules. In this study, we addressed these points to ensure that the introduced parts allow the correct distance and orientation between the donor and the acceptor molecule before and after VEGF binding. Therefore, ten eBRET2 VEGF biosensors with different parts of the ligand binding domains of the VEGF receptors 1 and 2 were designed and their performance analysed.

Two filter luminescence measurements of the biosensors after incubation with VEGF showed that in all ten biosensors the energy transfer is possible (Fig 3D). Interestingly, only few biosensors showed a relevant change in ΔBR after VEGF binding, namely Re02, Re06, Re07 and Re09 (Fig 4). It remains unclear, whether the differences result from varying affinities of the binding domains to VEGF, whether there is a sterical inhibition of the VEGF binding or whether the sterical structure does not allow a conformational change for the correct orientation of the donor and the acceptor molecule for optimal energy transfer.

One factor that could influence binding of VEGF and/or intramolecular mobility of the biosensor is the variance in the length of the different VEGF binding domains. Single domains have a size around 200 base pairs (bp), two domains are approximately 500 bp in size, and full length extracellular domains possess a length of 2000 bp. There seems to be a negative correlation between the length of the VEGF binding domains and the eBRET2 ratio, i.e. the longer the domain the lower the eBRET2 ratio (Fig 3C). Furthermore, the length of the VEGF binding domain not only has an influence on the eBRET2 performance, it is also negatively correlated with the luciferase activity (Fig 3A). This might be due to the correlation of the expression levels of the biosensors and the luciferase activity. The smaller the size of the biosensor, the higher the expression level of the protein and thus, a higher luciferase activity. Especially the biosensors Re04 and Re08 with the largest VEGF binding domain (entire molecule has a molecular weight of approximately 130–140 kDa) show a very weak luciferase activity and a low eBRET2 ratio. However, since the eBRET2 ratio is a concentration independent unit, the absolute biosensor concentration should not have a significant influence on the VEGF quantification. Nevertheless the biosensor concentration has an impact on the saturation of the biosensors with VEGF molecules. Higher biosensor concentrations lead to a later saturation and thus to a higher ΔBR, which was shown in Fig 4B. Consequently, smaller biosensors may have an advantage over larger biosensors because of their higher expression efficacy and the better eBRET2 performance.

Four biosensors showed significant changes in ΔBR after VEGF incubation. Re02 showed an increase of ΔBR in a range of 4 ng /ml VEGF, whereas Re06 detects VEGF at ~ 1 pg/ml, Re07 at ~ 4 ng/ml and Re09 at ~ 500 ng/ml VEGF (Fig 4). Each biosensor functions in a separate range for optimal VEGF detection. Measurements of the VEGF levels in anterior chamber fluid samples after limbal paracentesis showed a VEGF concentration of 68.0 ± 32.0 pg/ml [13] and the intravitreal VEGF concentration measured after vitrectomy was 71.0 ± 63.2 pg/ml [38]. Therefore, especially Re06 and Re07 with a very high sensitivity seem to be promising for VEGF quantification in vivo.

Another distinctive feature is that with the exception of Re08, all eBRET2 biosensors that contain the extracellular IgG like domain 3 from VEGF receptor 2 show the best results for VEGF quantification. This is in line with findings, which show domain 3 to be essential for the high affinity binding of VEGF-A [3–5]. The suboptimal performance of Re08 might rather correlate with the aforementioned drawback of the long VEGF binding domain. Furthermore, very interesting is the good performance in VEGF quantification of Re09, which contains a mixture of extracellular domain 2 of VEGF receptor 1 and extracellular domain 3 of VEGF receptor 2. This combination of VEGF binding domains is already known for high affinity binding of VEGF and is in use in the approved anti-VEGF molecule Aflibercept (Eylea^®^) [7, 8].

Besides affinity, the specificity of the eBRET2 VEGF biosensors is an important factor. The use of human VEGF receptors grant a high specificity to human VEGF. Admittedly, it is well known that the VEGF receptors 1 and 2 not only bind VEGF-A. VEGF receptor 1 is known to also bind VEGF-B and PLGF, whereas VEGF receptor 2 is able to bind proteolytically processed forms of VEGF-C and VEGF-D [5, 9, 39]. It is described, that Aflibercept not only binds to VEGF-A but also to VEGF-B, PlGF1, and PlGF-2 [7], so there is a high probability that the VEGF receptor based eBRET2 VEGF biosensors also bind other VEGF family members besides VEGF-A. There might be several purposes where this fact has to be kept in mind and if necessary has to be further investigated. Our point of interest is mainly the VEGF concentration in the eye before and after anti-VEGF treatment. Several studies demonstrated that in retinal and choroidal vascular diseases, especially the expression of VEGF-A is upregulated due to hypoxia, which is accountable for the pathological neovascularisation [13, 40, 41]. Admittedly, there is little evidence to suggest that other members of the VEGF family were involved in the pathogenesis of diabetic eye diseases [42], but it was also described that the intraocular concentration of the PLGF was decreased in AMD patients [43]. From these findings, we suggest that we will mainly measure VEGF-A in the eye and that the potential detection of other VEGF family members represents a negligible side effect.

In conclusion, we generated ten different eBRET2 VEGF biosensors of which four shows the ability to quantify VEGF in vitro. Every biosensor shows a unique detection range for VEGF quantification and especially Re06 and Re07 have a high sensitivity. Those results are very promising for future plans of expressing those biosensors immobilized in an implantable device for in vivo VEGF quantification in the eye. Mainly Re06 would be suitable for the detection of even low physiologic VEGF concentrations in the eye what would deliver a great advantage in the personalized and individualized treatment of patients.

## Supporting information

S1 Table PCR-Primer for the extracellular IgG like domains of the VEGF receptors one (R1) and two (R2). D2 = extracellular IgG like domain 2, D3 = extracellular IgG like domain 3, FL = full-length extracellular IgG like domain, f = forward, r = reverse. All Primer were phosphorylated (P) at their 5’-end for later ligation of the PCR products into the biosensor backbone vector.
(JPG)

S1 Fig GFP2 expression in HEK293-T cells. 24 h after transfection the activity of the biosensor component GFP2 was verified by fluorescence microscopy with the Keyence BZ-8000. Cells were washed once with PBS to reduce the background signal of the Medium. Images were recorded with an exposure time of < 1 s. Scale bar is 50 μm.
(TIF)

S2 Fig VEGF dependent Delta eBRET2 ratio. The Biosensor concentrations in the cell lysates were adjusted to equal amounts on the basis of Rluc8 expression. 20μl of these lysates were incubated with 10μl serially diluted recombinant human VEGF. After 24h incubation BRET2 Ratios were measured via two filter luminescence scan and Delta eBRET2 Ratios were calculated.
(TIF)

S1 Raw Images (PDF)
