# Supplementary figures and images for: Creation of different bioluminescence resonance energy transfer based biosensors with high affinity to VEGF
Source: PLoS One. 2020 Mar 26;15(3):e0230344. doi: 10.1371/journal.pone.0230344 (PMC7098639; doi:10.1371/journal.pone.0230344)

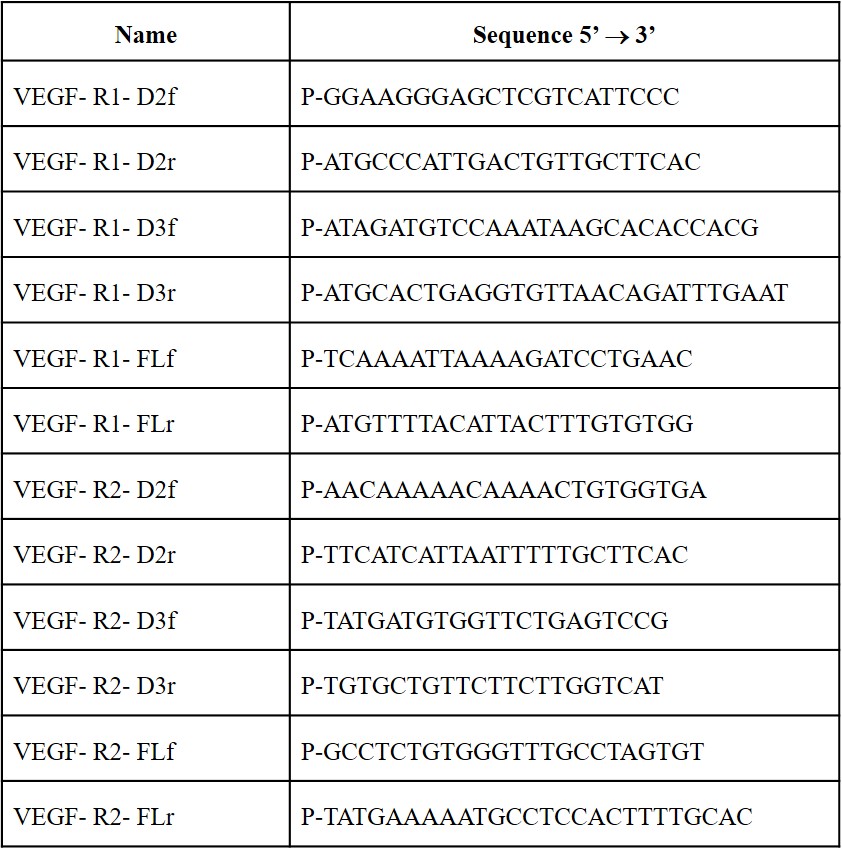

Supplement: S1 Table — D2 = extracellular IgG like domain 2, D3 = extracellular IgG like domain 3, FL = full-length extracellular IgG like domain, f = forward, r = reverse. All Primer were phosphorylated (P) at their 5’-end for later ligation of the PCR products into the biosensor backbone vector. (JPG) [file pone.0230344.s001.jpg]

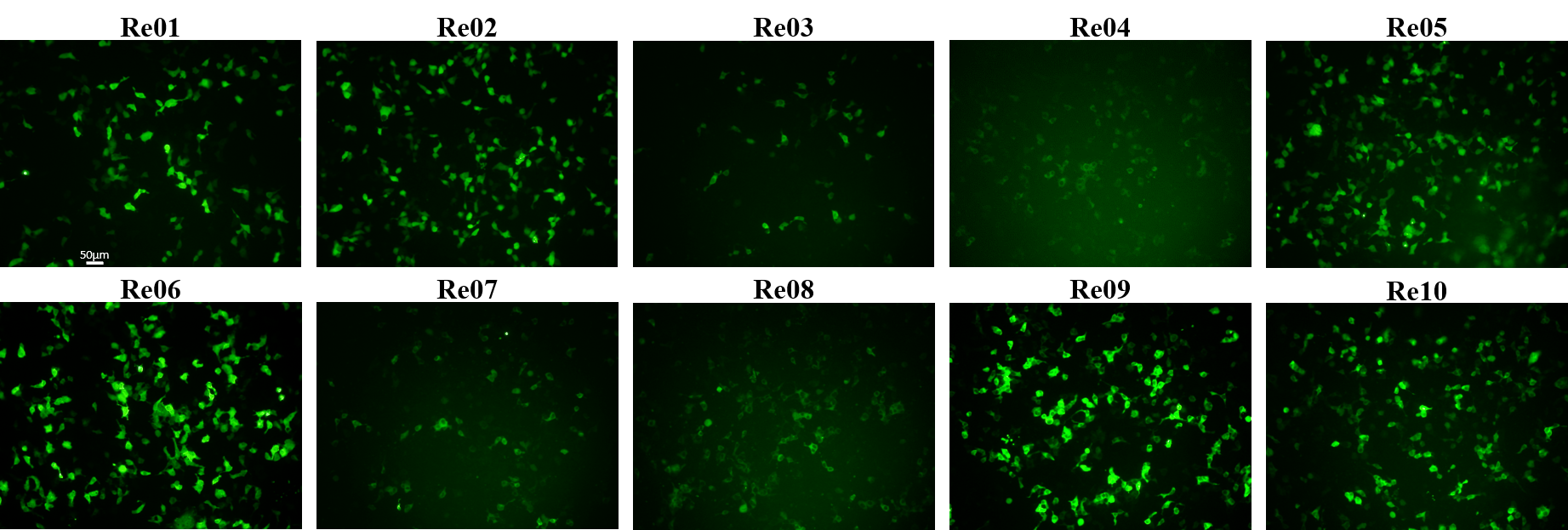

Supplement: S1 Fig — 24 h after transfection the activity of the biosensor component GFP2 was verified by fluorescence microscopy with the Keyence BZ-8000. Cells were washed once with PBS to reduce the background signal of the Medium. Images were recorded with an exposure time of < 1 s. Scale bar is 50 μm. (TIF) [file pone.0230344.s002.tif]

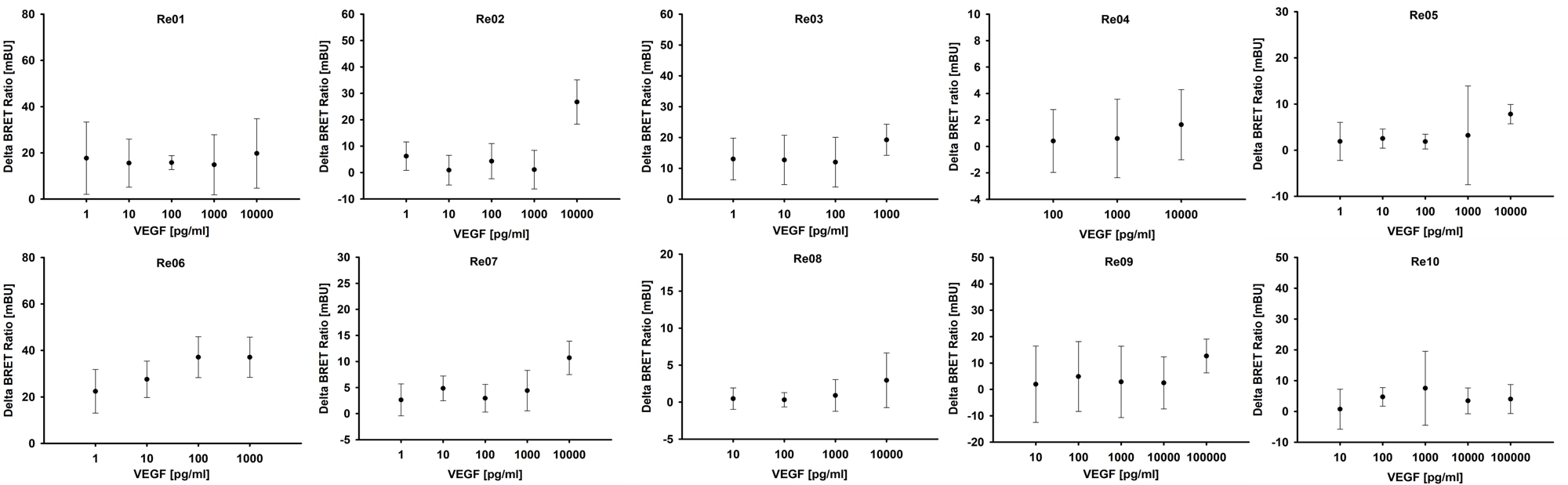

Supplement: S2 Fig — The Biosensor concentrations in the cell lysates were adjusted to equal amounts on the basis of Rluc8 expression. 20μl of these lysates were incubated with 10μl serially diluted recombinant human VEGF. After 24h incubation BRET2 Ratios were measured via two filter luminescence scan and Delta eBRET2 Ratios were calculated. (TIF) [file pone.0230344.s003.tif]
