# Supplementary material for: Creation of different bioluminescence resonance energy transfer based biosensors with high affinity to VEGF
Source: PLoS One. 2020 Mar 26;15(3):e0230344. doi: 10.1371/journal.pone.0230344 (PMC7098639; doi:10.1371/journal.pone.0230344)

- Figure 2 D, Western Blot, upper part
- Cell lysates were used in SDS-Page to separate Proteins by size. Afterwards Proteins were transferred onto a nitrocellulose membrane via semidry blotting
- As first antibody the polyclonal antibody rabbit anti Rluc8 was used.
- The second antibody was a goat anti rabbit IgG conjugated with horse radish peroxidase (HRP). For HRP detection the GE Healthcare Amersham™ ECL™ Western Blotting-detection reagent and the chemiluminescent films GE Healthcare™ Amersham™ Hyperfilm were used.

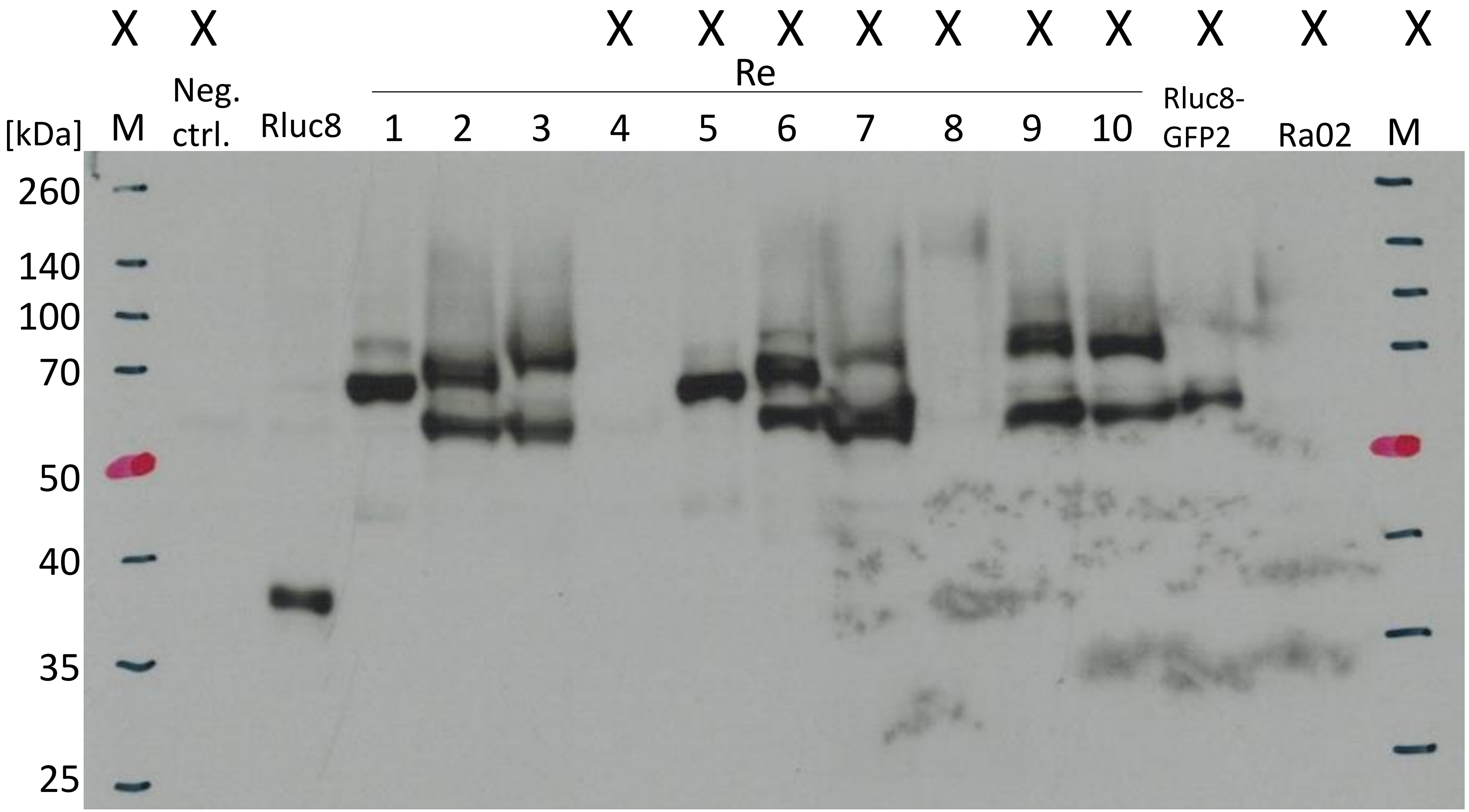

- Figure 2 D, Western Blot, lower part
- Cell lysates were used in SDS-Page to separate Proteins by size. Afterwards Proteins were transferred onto a nitrocellulose membrane via semidry blotting
- As first antibody the polyclonal antibody rabbit anti GAPDH was used.
- The second antibody was a goat anti rabbit IgG conjugated with horse radish peroxidase (HRP). For HRP detection the GE Healthcare Amersham™ ECL™ Western Blotting-detection reagent and the chemiluminescent films GE Healthcare™ Amersham™ Hyperfilm were used.

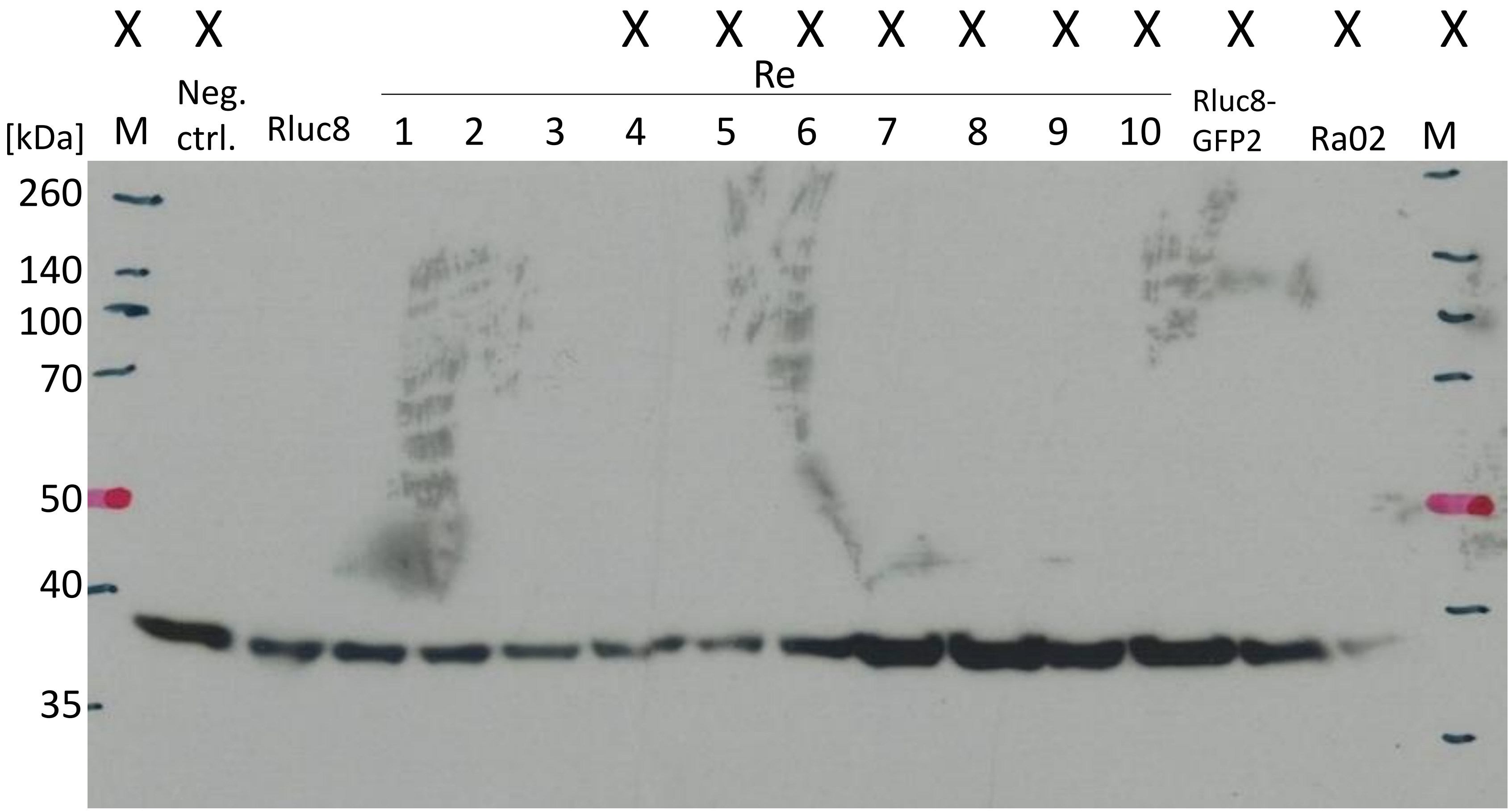

- Figure 2 F, Western Blot, upper part
- After deletion of the donor splice site the cell lysates were used in SDS-Page to separate Proteins by size. Afterwards Proteins were transferred onto a nitrocellulose membrane via semidry blotting
- As first antibody the polyclonal antibody rabbit anti Rluc8 was used.
- The second antibody was a goat anti rabbit IgG conjugated with horse radish peroxidase (HRP). For HRP detection the GE Healthcare Amersham™ ECL™ Western Blotting-detection reagent and the chemiluminescent films GE Healthcare™ Amersham™ Hyperfilm were used.

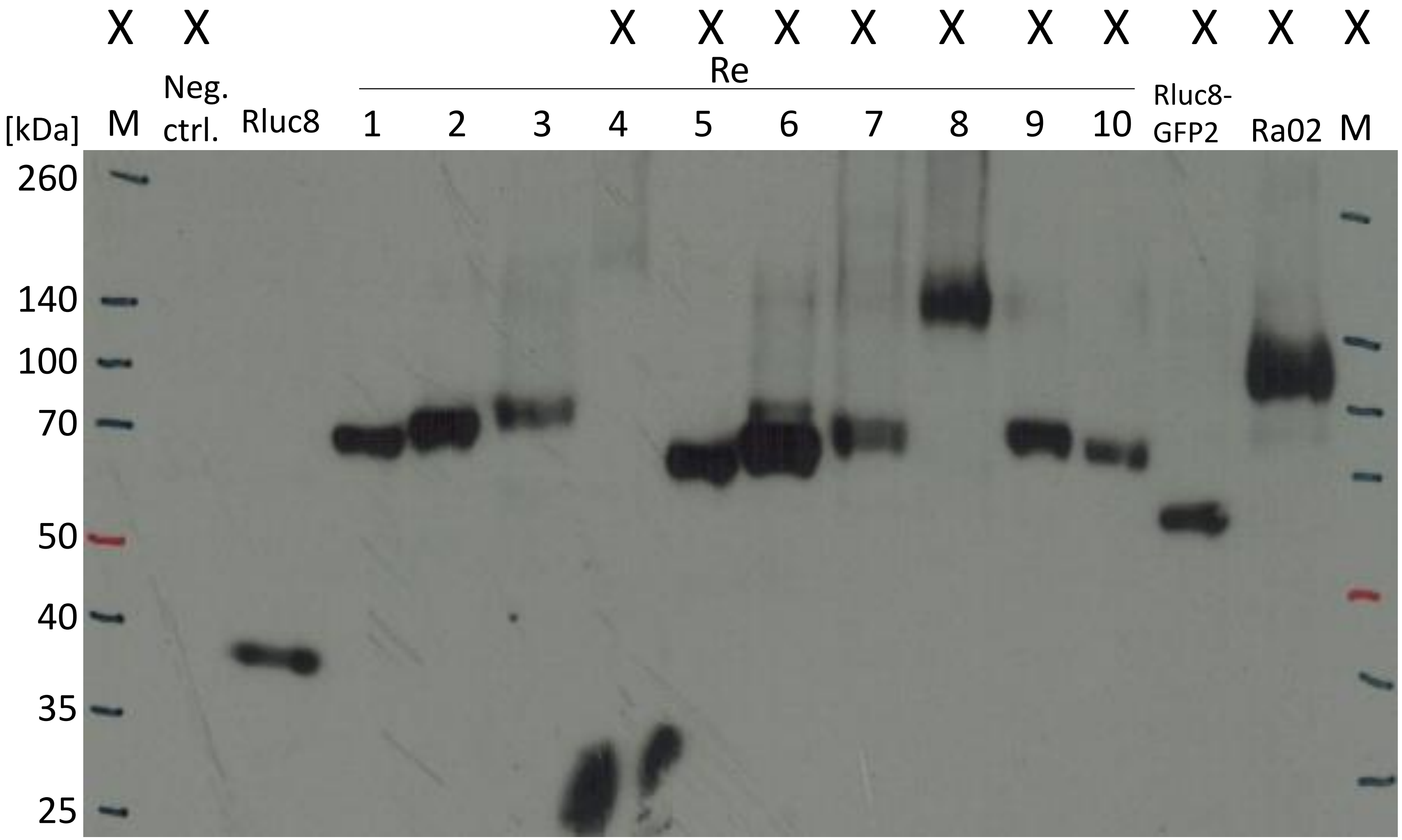

- Figure 2 F, Western Blot, lower part
- After deletion of the donor splice site the cell lysates were used in SDS-Page to separate Proteins by size. Afterwards Proteins were transferred onto a nitrocellulose membrane via semidry blotting
- As first antibody the polyclonal antibody rabbit anti GAPDH was used.
- The second antibody was a goat anti rabbit IgG conjugated with horse radish peroxidase (HRP). For HRP detection the GE Healthcare Amersham™ ECL™ Western Blotting-detection reagent and the chemiluminescent films GE Healthcare™ Amersham™ Hyperfilm were used.

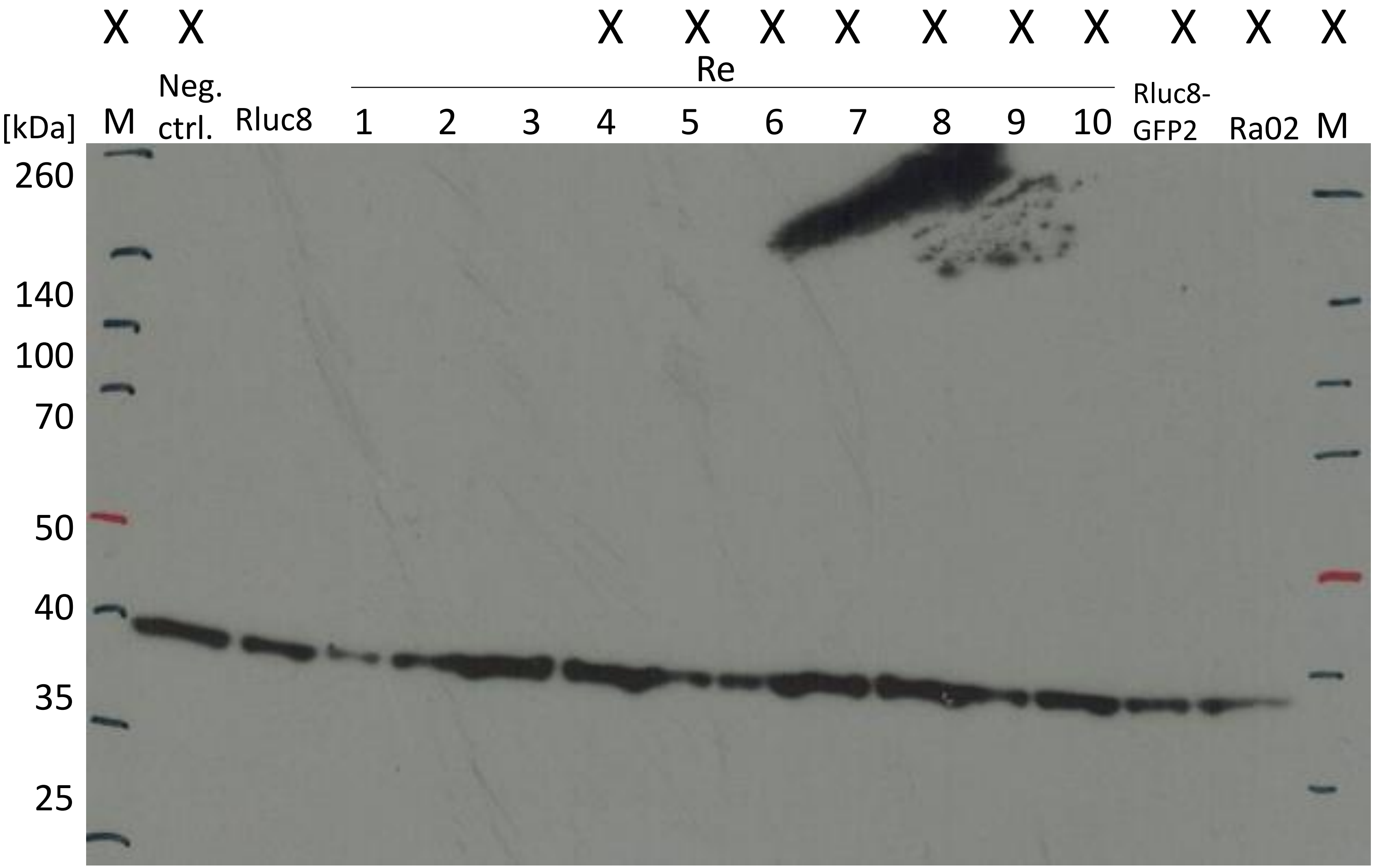

- Figure 3 B, Western Blot, upper part
- After deletion of the donor splice site the cell lysates were used in SDS-Page to separate Proteins by size. Afterwards Proteins were transferred onto a nitrocellulose membrane via semidry blotting
- As first antibody the polyclonal antibody rabbit anti Rluc8 was used.
- The second antibody was a goat anti rabbit IgG conjugated with horse radish peroxidase (HRP). For HRP detection the GE Healthcare Amersham™ ECL™ Western Blotting-detection reagent and the chemiluminescent films GE Healthcare™ Amersham™ Hyperfilm were used.

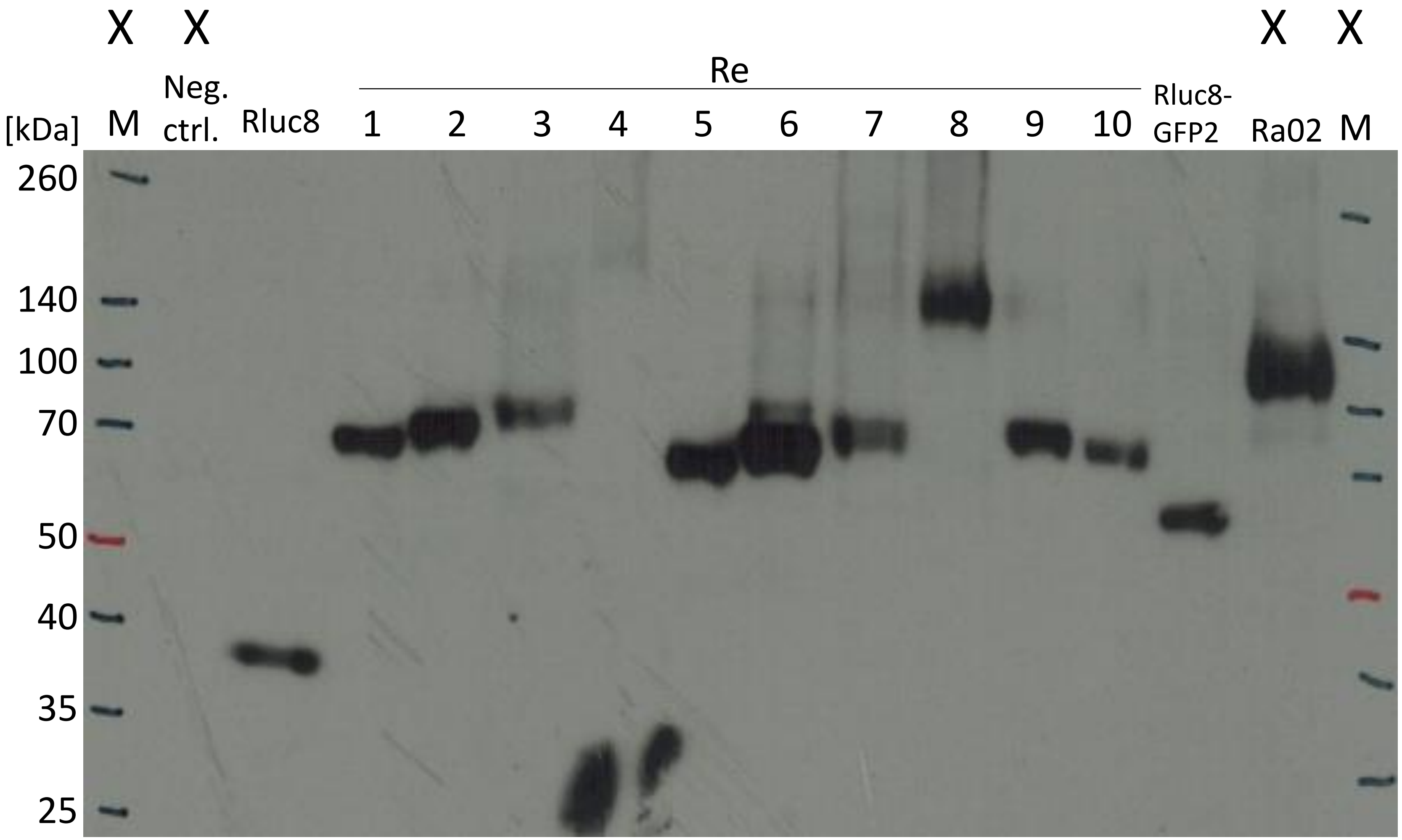

- Figure 3 B , Western Blot, lower part
- After deletion of the donor splice site the cell lysates were used in SDS-Page to separate Proteins by size. Afterwards Proteins were transferred onto a nitrocellulose membrane via semidry blotting
- As first antibody the polyclonal antibody rabbit anti GAPDH was used.
- The second antibody was a goat anti rabbit IgG conjugated with horse radish peroxidase (HRP). For HRP detection the GE Healthcare Amersham™ ECL™ Western Blotting-detection reagent and the chemiluminescent films GE Healthcare™ Amersham™ Hyperfilm were used.

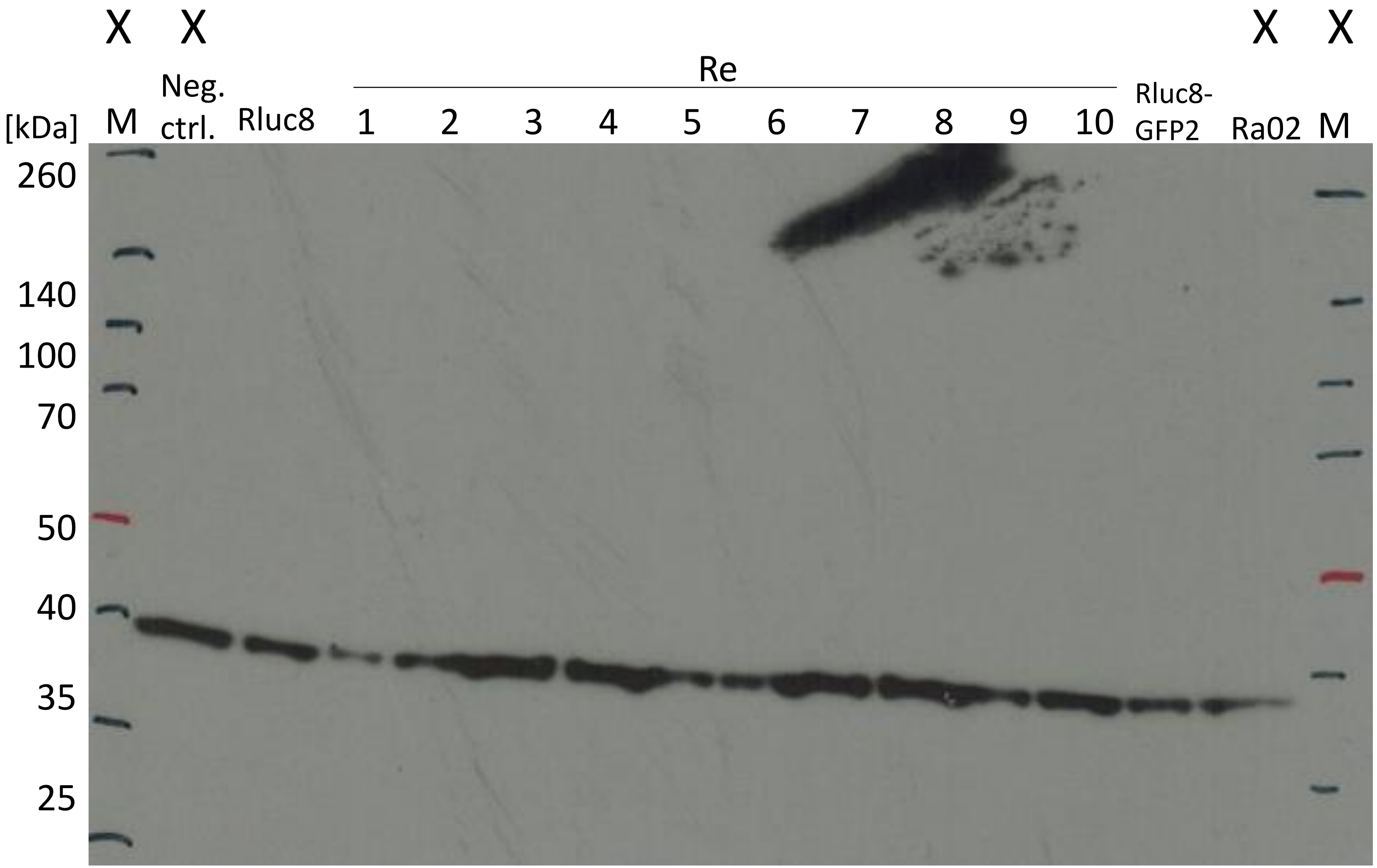

Supplement: S1 Raw Images — (PDF) [file pone.0230344.s004.pdf]
